# Altered ruminal microbiome tryptophan metabolism and their derived 3-indoleacetic acid inhibit ruminal inflammation in subacute ruminal acidosis goats

**DOI:** 10.1186/s40168-025-02202-x

**Published:** 2025-10-23

**Authors:** Xiaodong Chen, Jingyi Xu, Lei Zhang, Bingxuan Xie, Jianrong Ren, Jinghui He, Tao Liu, Qingqing Liu, Yachen Dong, Xiaolong He, Junhu Yao, Shengru Wu

**Affiliations:** 1 College of Animal Science and Technology, Northwest A&F University, Yangling , Shaanxi, 712100 China; 2 Key Laboratory of Livestock Biology, Northwest A&F University, Yangling , Shaanxi, 712100 China; 3 College of Animal Science and Technology, Ningxia University, Yinchuan, 750021 China; 4 Shanghai Majorbio Biopharm Technology Co. Ltd, Shanghai, 201318 China

**Keywords:** Dairy goats, Subacute rumen acidosis, Epithelial inflammation, Ruminal microbiome, Tryptophan metabolism

## Abstract

**Background:**

Subacute ruminal acidosis (SARA) is a digestive disorder that often severely jeopardizes the health and lactation performance of ruminants fed a high-energy diet. Different dairy ruminants exhibit varying degrees of inflammation accompanied by variations in the rumen microbiota when SARA occurs. Our understanding of the occurrence of SARA and varying degrees of rumen epithelial inflammation is lacking. Hence, we performed rumen metagenomic, metagenome-assembled genome and metabolomic analyses, with transcriptome and single-nucleus RNA sequence analyses, to explore the microbial mechanism of SARA occurrence and different degrees of inflammation.

**Results:**

A total of 36 goats fed two diets with gradually increasing levels of rumen-degradable starch (RDS) were included in this study, and SARA goats fed 70% concentrate diets supplemented with whole corn (HGW-SARA) and SARA goats fed 70% concentrate diets supplemented with crushed corn (HGC-SARA) were identified. Moreover, 11 goats fed a control basal diet, named LGW-CON, were also included. Compared with those in the LGW-CON group, the rumen fermentation capacity was enhanced, accompanied by ruminal epithelial and systemic inflammation, in goats from HGW-SARA and HGC-SARA. Between them, HGC-SARA goats presented less inflammation. Notably, the ruminal inflammation-related pathways were increased only in the HGW-SARA group but not in the HGC-SARA group. Metagenomic analysis revealed that the β diversity of SARA goats was significantly different from that of LGW-CON goats. *Ruminococcus* significantly increased in both SARA groups, whereas *Prevotella* and *Bacteroidales* significantly decreased, which was accompanied by a decrease in cellulose and hemicellulose enzymes and an increase in lysozymes and lipopolysaccharide synthesis enzymes. Multi-omics analysis of the ruminal contents and tissues suggested that epithelial inflammation was caused by disturbed ruminal microbiome-induced Th17 cell differentiation and IL-17 signalling pathway activation. Comparative analyses between the HGW-SARA and HGC-SARA groups highlighted the importance of *Selenomonas* and *Bifidobacterium*, as well as bacterial tryptophan metabolism, in the production of 3-indoleacetic acid, which mitigated ruminal epithelial inflammation by modulating Th17 cells and inhibiting IL-17 signalling. Ruminal microbiota transplantation from HGW-SARA goats to healthy dairy goats and mice revealed the role of microbes in epithelial inflammation. Additionally, 3-indoleacetic acid supplementation reduced rumen inflammation and the IL-17 concentration in the serum, improved VFAs absorption, and enhanced milk production.

**Conclusions:**

This study unveiled that after SARA was induced by high-concentrate feeding, the rumen homeostasis was disrupted, and rumen fiber degradation capacity of dairy goats decreased, but the LPS synthesis capacity increased, and inflammation of the rumen epithelium was observed. However, the ruminal microbial species from the *Bifidobacterium* and *Selenomonas* genera and bacterial 3-indole acetic acid are pivotal in mitigating ruminal epithelial inflammation during SARA in dairy goats. This could potentially be attributed to the modulation of ruminal Th17 cell proportions and the inhibition of IL-17 signalling pathways.

Video Abstract

**Supplementary Information:**

The online version contains supplementary material available at 10.1186/s40168-025-02202-x.

## Introduction

Given the growing world population and high rates of malnutrition, food security has become an increasingly critical issue. Ruminant products provide nutrient-dense food sources, but the increasing demand for these products is likely to soon outweigh the available resources [1]. Dairy animals are commonly fed high-concentrate diets to increase milk production; nonetheless, these diets can cause some animals to develop subacute ruminal acidosis (SARA), resulting in poor health and reduced milk production [2–5]. However, these previous studies focused mainly on bacterial composition changes and their correlations with the above phenotype changes related to SARA. Identifying metagenome alterations will help to better understand microbial roles during the occurrence of SARA.

Previous studies have shown that decreased ruminal pH can induce ruminal gram-negative bacterial lysis and the release of LPS, leading to ruminal epithelium inflammation through the activation of the TLR/MyD88-NFκB pathway when SARA occurs [6–8]. Furthermore, several studies have indicated that the development of gut inflammation in mammals can be mediated by T helper cells [9, 10]; among them, Th17 cells are the most critical Th cells for mediating gastrointestinal inflammatory responses [11, 12]. Interleukin-17 plays a critical role in promoting inflammatory responses by inducing the expression of pro-inflammatory cytokines and chemokines, contributing to the amplification of inflammation. The levels of IL-17 secreted by Th17 cells are positively correlated with gastrointestinal inflammation [13–15]. Moreover, several recent studies have suggested that the IL-17 signalling pathways are significantly enhanced in SARA cows [2, 16]. However, under conditions of low pH and increased LPS content, some individual dairy cows or goats do not exhibit ruminal inflammation [17, 18].

Hence, in this study, we aimed to use integrated meta-omics based on metagenome or single-nucleus RNA sequencing (snRNA-seq) technologies to address the following research questions: (1) how do ruminal metagenome function alter in response to a high-concentration diet and SARA occurrence? (2) How are rumen epithelial cell subtypes, especially immune cell composition and gene expression changes, related to the occurrence and inhibition of rumen epithelial inflammation? (3) Are the individualized ruminal microbiome involved in regulating different ruminal inflammation responses? (4) What are the interactions among the ruminal microbiome, rumen epithelial cells and immune cells, and do these interactions coregulate ruminal inflammation when goats are fed high-concentrate feed?

## Methods

### Dairy goats feeding experiment and study design

All the experimental designs and protocols used in the present study were approved by the Institutional Animal Care and Use Committee (IACUC) of Northwest A&F University (Shaanxi, China; approval number: NWAFU-DK-2022155).

Forty-seven healthy, multiparous female dairy goats (2 to 3 years, ~ 40 kg) with ruminal fistulas were used. Eleven dairy goats, were fed a basal diet (30% concentrate, DM basis, named as LGW diet) (Table S1). The other 36 dairy goats were subsequently fed a high-concentrate diet supplemented with whole corn (70% concentrate, DM basis, named as HGW diet and referred to as the low-rumen-degradable-starch diet) (Table S1). A total of 2 kg of fresh TMR experimental diet (under restricted feeding condition ensuring that all dairy goats consumed their entire ration daily without any leftover feed) was fed to each goat twice daily at 08:00 and 17:00. On the 28th and 35th days, the pH of the ruminal fluid was measured for 14 h with a mobile pH meter (HI 9024 C; RI, USA). The HGW feeding goats (*n* = 8), whose pH was lower than 5.8 for more than 3 h, were determined to be high-grain-whole-corn SARA (HGW-SARA) goats. The remaining dairy goats, selecting 5 of them to keep feeding HGW diet, the others were further fed a high-rumen-degradable-starch diet with crushed corn for another 28 days (70% concentrate, DM basis, named the HGC diet) (Table S1). Similarly, on the 56th and 63rd days of the entire trial period, the ruminal pH of all the goats was determined. When fed the HGC diet, dairy goats (*n* = 7), whose pH was lower than 5.8 for more than 3 h, were determined to be high-grain-crushed-corn SARA (HGC-SARA) goats.

### Ruminal microbiota transplantation (RMT) from donor SARA goats to healthy recipient goats

Another eight healthy dairy goats with rumen fistulas were fed a basal diet (30% concentrate, DM basis) (Table S1). On the slaughter days, the ruminal fluid of the 3 donor HGW-SARA goats was collected and then transplanted to another 3 healthy dairy goats with ruminal fistulas after the ruminal fluid content was removed. The detailed steps were performed in accordance with the methods of Zhou [19]. This group of recipient goats was subsequently named the CON + SARA group, and another 5 healthy dairy goats were named the CON group. After transplantation, the goats in the CON and CON + SARA groups were further fed a normal concentration of feed (50% concentrate, DM basis) for 2 weeks (Table S2). These 8 goats were subsequently slaughtered for sample collection.

### Generated SARA model of dairy goats and gavaged 3-indoleacetic acid

The 24 dairy goats were fed the same high-grain diet with a concentrate:forage ratio of 6:4 (Table S3) and divided into high rumen-degradable-starch control (HRDS-C) and high rumen-degradable-starch with 3-indoleacetic acid (HRDS-IA) groups. The twelve goats in HRDS-IA were fed 2.5 g/day 3-indoleacetic acid (Sigma‒Aldrich, China), which was mixed into the concentrates. The experimental period lasted 6 weeks, and the rumen fluid and plasma samples were collected at 7:00, 9:00, 11:00, 17:00, and 19:00 on the 42nd day.

### Sample collection of slaughtered goats

Before slaughter, 20 mL of blood was collected from the jugular vein and anticoagulated with heparin sodium. After slaughtering, 200 mL of rumen fluid was collected. Furthermore, approximately 1 cm^2^ of epithelial tissue from the dorsal rumen at a similar position was collected from each goat. The other epithelial tissues of the rumen were collected in 2 × 2 cm^2^ pieces and fixed in optimum cutting temperature (OCT) compound.

### RMT from goats to mice and sample collection of mouse recipients

RMT from goats to mice was performed in accordance with our previously published methods [20]. On slaughter days, the ruminal fluid of the slaughtered HGW-SARA goats (*n* = 5) and LGW-CON goats (*n* = 5) was collected, placed inside a sterile and anaerobic collection tube, and then centrifuged at 6000 × *g* for 15 min. The precipitate without the supernatant was resuspended in 1 × PBS, and then, the resulting suspensions were transferred directly to the recipient mice. Ten male Kunming (KM) mice underwent a 10-day adaptation period and were then treated with antibiotics for 3 weeks dissolved in drinking water and for 3 days via gavage (Fig. S1 and Table S4). After a 24-h antibiotic-free period, the mice were infused by intragastric gavage with 0.3 mL of mixed rumen fluid derived from LGW-CON goats (called CON) or HGW-SARA goats (called CON + SARA) for 3 days. After being fed for 10 days, all the mice were sacrificed and weighed, and colonic tissues were collected for further RNA extraction and inflammation-related gene expression detection.

### Rumen and plasma samples analysis

The rumen fluid was centrifuged at 13,000 × *g* for 10 min. We analyzed the concentration of VFAs in the rumen fluid and plasma and the concentration of lactate in the rumen fluid, which were separated and quantified with an Agilent 7820 GC system, as previously described [21, 22]. The ruminal NH₃-N levels were measured via the phenol‒sodium hypochlorite colorimetric method. Lipopolysaccharide (LPS) was quantified via a Limulus amoebocyte lysate (LAL) assay, and the level of lipopolysaccharide binding protein (LBP) was determined via an indirect competitive enzyme-linked immunosorbent assay [23, 24]. The levels of the rumen and plasma inflammation-related proteins IL-1β, TLR-4, LBP, IL-6, IL-10, TNF-α, and IL-17 were measured with commercial ELISA kits (COIBO BIO, Shanghai, China).

### Histological analysis and immunofluorescence

Rumen tissues were fixed in 4% paraformaldehyde, sectioned (4 μm), and stained with H&E. The immunofluorescence measurements were conducted as previously described [25]. Immunofluorescence was performed using anti-IL-17A antibodies, with nuclei stained by DAPI. Images were analyzed via ImageJ.

### Quantitative real-time PCR

Total RNA extraction was performed via TRIzol reagent (Carlsbad, USA), and the RNA was reverse transcribed into cDNA following the standard protocol provided with the PrimeScript® RT Kit (Takara, China). Quantitative real-time polymerase chain reaction (qRT‒PCR) assays were executed with SYBR® Premix Pro TaqTM II (Takara, China) on a real-time fluorescence quantitative thermal cycler (Light Cycler 9,603,030,973). The specific primer pairs utilized for the target genes are listed in Table S5. Gene expression levels were analyzed via the 2^−ΔΔCt^ method for relative quantification [26].

### Metagenomic analysis

Total metagenomic DNA was extracted from the rumen fluid samples via the E.Z.N.A.® Soil DNA Kit (Omega Biotek, Norcross, GA, USA), with repeated bead beating and after concentration, purification and integrity verification, the DNA was fragmented to ~ 400 bp. After bridge PCR amplification, metagenomic sequencing was performed via an Illumina NovaSeq/HiSeq Xten (Illumina, USA) sequencing platform.

Paired-end Illumina reads underwent adaptor trimming and filtering of low-quality sequences utilizing fastp [27] (version 0.20.0) and Trimmomatic (version 0.39). The reads were aligned to the reference genome of Capra hircus via BWA [28, 29] (version 0.7.9a), and assembled with MEGAHIT [30] (version 1.1.2). Contigs reaching or exceeding 300 bp in length were retained as the final assembly output. Open reading frames (ORFs) were inferred from assembled contigs utilizing MetaGene. ORFs extending to or beyond 100 base pairs were extracted and translated into amino acid sequences to compile a nonredundant gene catalogue via CD-HIT [31] (version 4.6.1 Postquality control reads were mapped to the nonredundant gene catalogue with 95% identity via SOAPaligner (version 2.21), evaluating quantification of gene abundance within individual samples. The total gene abundance of each sample was normalized.

For taxonomic classification, representative sequences of the nonredundant gene catalogue were aligned to the NCBI NR database via Diamond [32] (version 0.8.35). Functional annotations via the Kyoto Encyclopedia of Genes and Genomes (KEGG) were carried out with Diamond [32] against the KEGG database. Carbohydrate-active enzymes (CAZymes) were annotated by subjecting the catalogue to hmmscan analysis against the CAZy database. The relative contribution of species to the microbial function were calculated using the method described by Zhang et al. [33].

### Metagenomic binning

The metagenomic assembled contigs were obtained. The contig sequences ≥ 1000 bp were filtered via the case tools Metabat [34] (Version 2.12.1), CONCOCT [35] (Version 0.5.0), and Maxbin [36] (Version 2.2.5) to conduct single-sample binning. The bins of different software programs were merged via DAS_Tool [37] Version 1.1.0), and the bins were regenerated. RefineM (Version 0.0.24) was used to purify bins and obtain bins that were redefined as metagenome-assembled genomes (MAGs). All binned MAGs were clustered together, and dRep (Version 2.2.9) was used to cluster the bins to remove duplicate MAGs under the thresholds of ANI ≥ 99% and genome overlap ≥ 10%. In accordance with the CheckM [38] (Version 1.0.12) quality evaluation standard, the nonredundant MAGs were selected as having medium quality (completeness ≥ 50% and contamination < 10%) and were further analyzed. Taxonomic assignment was performed via GTDB-tk [39].

### Metabolomic analysis

Rumen fluid metabolites and a pooled quality control (QC) sample were analyzed by liquid chromatography‒mass spectrometry (LC‒MS). Differentially abundant metabolites were summarized by PLS-DA (VIP ≥ 1, fold change ≥ 2 or ≤ 0.5, *q* < 0.05) and mapped to their biochemical pathways through metabolic enrichment and pathway analysis via the KEGG database.

### Transcriptomic analysis

Total RNA was extracted from the tissue via TRIzol® Reagent according to the manufacturer’s instructions (Invitrogen) The construction of transcriptome libraries and sequencing methods refer to Liu et al. [40]. The raw paired-end reads were trimmed and quality controlled by SeqPrep and Sickle with default parameters. The clean reads were subsequently separately aligned to the reference genome via HISAT2 [41]. The mapped reads of each sample were assembled via StringTie via a reference-based approach [42].

The expression level of each transcript was calculated according to the transcripts per million reads (TPM) method. RSEM [43] was used to quantify gene abundances. Essentially, differential expression analysis was performed via DESeq2 [44], and DEGs with |log2FC|> 1 and a *P* value ≤ 0.05 were considered to be significantly DEGs. KEGG functional enrichment analyses were performed via KOBAS with threshold as BH-corrected *P* values ≤ 0.05. Furthermore, the gene expression of differential KEGG pathways was further identified via gene set enrichment analysis (GSEA) [45].

### Single-nucleus RNA analysis

The specific method was described in a previous study [46]. Briefly, the sample was incubated on ice, filtered, and centrifuged. The isolated nuclei were resuspended, filtered and counted. A final concentration of 1000 nuclei per microliter was used for loading on a 10 × channel. The gel beads-in-emulsion (GEM) mixture was exposed to cell lysis buffer, and polyadenylated RNA molecules were retrieved for reverse transcription to cDNA. The library was constructed, quantified and then sequenced to obtain 150 bp paired-end reads.

The reads were processed via the Cell Ranger workflow. Clean reads were aligned to *Capra_hircus* ARS1.2 via the STAR algorithm [47]. A gene‒barcode matrix was then imported into the Seurat R toolkit for quality control. The clusters are visualized on a 2D map produced with t-distributed stochastic neighbor embedding (t-SNE) [48]. The cell cluster identity was assigned by manual annotation on the basis of the expression of known marker genes. DEG (differentially expressed gene) identification was performed with |log2FC|> 0.25 and a Q value < = 0.05 via the function FindMarkers in Seurat via a likelihood ratio test. KEGG enrichment analysis was performed to identify which DEGs were significantly enriched in KEGG pathways at a Bonferroni-corrected *P* value ≤ 0.05.

To identify DEGs between two different samples or clusters, the function FindMarkers in Seurat was used, and a likelihood ratio test was performed. Essentially, DEGs with |log2FC|> 0.25 and a *Q* value < = 0.05 were considered significantly differentially expressed genes.

### Statistical analyses

The statistical analyses were conducted in IBM SPSS Statistics 27 via the mean ± SEM and one-way ANOVA followed by the least significant difference (LSD) test. For data that were not normally distributed, the Kruskal‒Wallis H test was used to compare the differences. *P < 0.05, **P < 0.01, and ***P < 0.001 indicate significance and were visualized with GraphPad Prism 9.5. The correlation analysis was performed in R studio with the R package ComplexHeatmap. The structural equation model was built via the R package lavaan, with data dimension reduction handled via the vegan package [49]. Data normalization was performed with R’s scale function. The variance‒covariance matrix was estimated via maximum likelihood, and model fit was assessed via the *P* value, comparative fit index (CFI), goodness-of-fit index (GFI), and degrees of freedom (DF).

## Results

### SARA affected ruminal fermentation and caused rumen epithelial inflammation

**Fig. 1:**
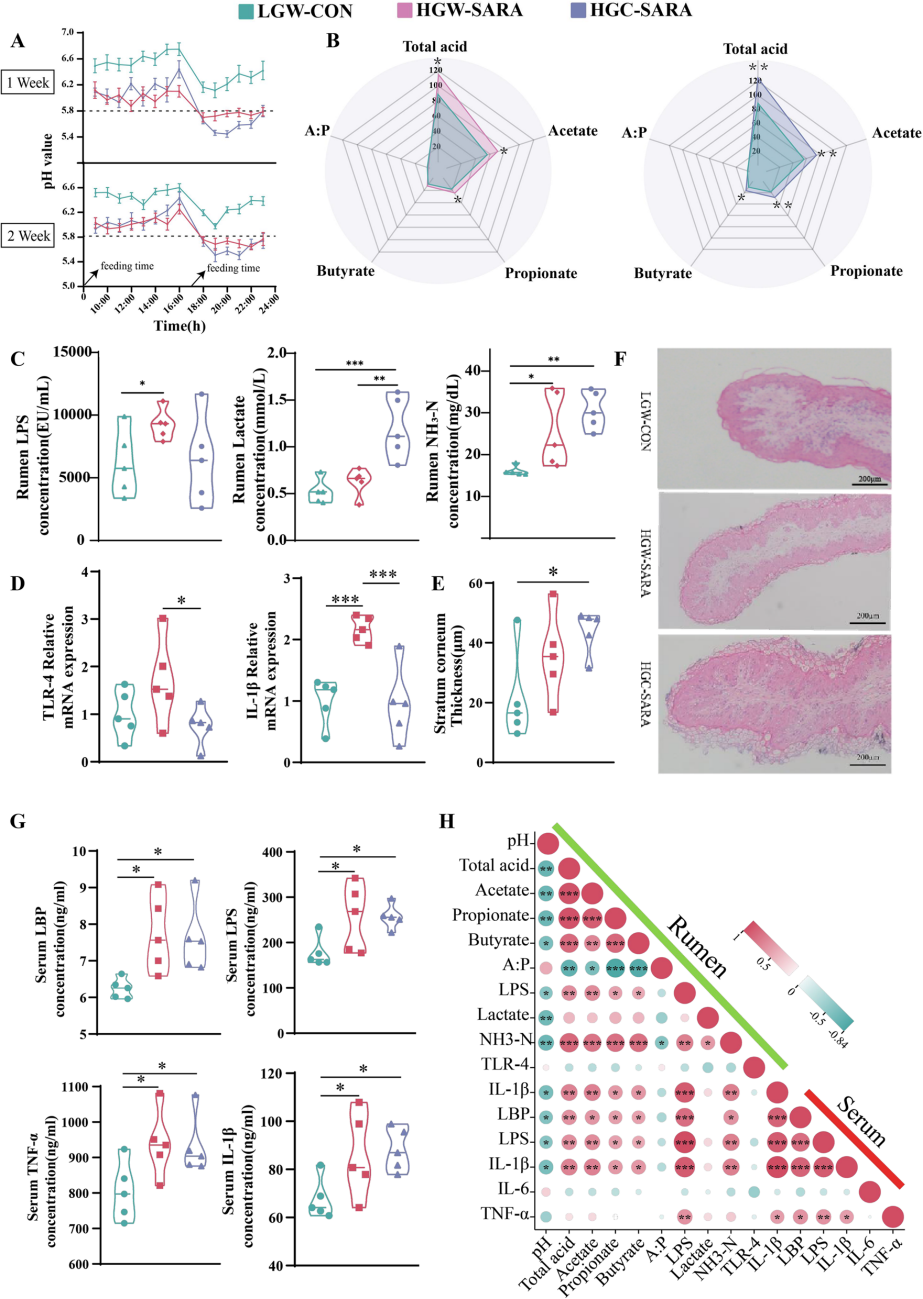
Fermentation and inflammatory parameters of the different SARA phenotypes associated with various diets. **A** The dynamic pH variation of dairy goats’ ruminal fluids 14 h after morning feeding on the 56th and 63rd days. **B** Differential VFAs concentrations of ruminal fluids between LGW-CON and HGW-SARA and between LGW-CON and HGC-SARA. **C** Different concentrations of LPS, lactate and NH_3_-N in the ruminal fluid. **D** The relative mRNA expression of TLR-4 and IL-1β in the ruminal epithelium. **E**–**F** The thickness of the stratum corneum and the morphology of the ruminal epithelial papilla. **G** Concentrations of LBP, LPS, TNF-α, and IL-1β in the serum. **H** Spearman correlation analysis of fermentation and inflammatory parameters in both the rumen and serum. Repeated measures in a general linear model for pH and one-way ANOVA for other variables, followed by post hoc LSD and DUNCAN tests, were used to conduct the statistical analysis. *P < 0.05, **P < 0.01, ***P < 0.001 indicate significance

On days 56 and 63 of the trial period, the pH level in the rumen of goats in the HGW-SARA and HGC-SARA groups remained below 5.8 for more than 6 h on each of those days (Fig. S1). This exceeded the critical threshold for diagnosing SARA (Fig. 1A). Compared with those in the LGW-CON group, the concentrations of ruminal total VFAs, acetate, propionate, and NH_3_-N were significantly greater in goats from the HGW-SARA and HGC-SARA groups (Fig. 1B, 1C), but the ruminal butyrate and lactate concentrations were significantly increased only in the HGC-SARA group, whereas the ruminal LPS concentration was significantly increased only in the HGW-SARA group (Fig. 1B, 1C). Furthermore, significantly greater gene expression of TLR-4 and IL-1β was detected in the HGW-SARA groups than in the LGW-CON and HGC-SARA groups (Fig. 1D), whereas the stratum corneum thickness of the rumen epithelium was significantly greater in the HGC-SARA groups than in the LGC-CON group (Fig. 1E, 1F).

In addition to the epithelial injury and inflammation in these 2 SARA groups, systemic inflammation may have occurred, which can be identified from the significantly increased serum LPS, LPS binding protein (LBP), TNF-α, IL-1β, and IL-6 concentrations in the HGW-SARA and HGC-SARA groups compared with those in the LGW-CON group (Fig. 1G). Owing to the effects of restricted feeding, the final body weights of all the goats slightly increased but not significantly differed among the 3 groups, and only the body weight gain of the CON group was significantly lower than that of the HGC-SARA group (Table S6). Further association analysis indicated that ruminal fermentation indices were negatively associated with the ruminal pH and that the ruminal pH was further negatively associated with the ruminal LPS concentration. Furthermore, ruminal LPS was positively associated with the serum LPS concentration and was positively associated with pro-inflammatory factors (Fig. 1H).

### Different responses of the rumen epithelium to SARA between HGW-SARA and HGC-SARA

The ruminal epithelial transcriptomes revealed that more infection- and inflammation-related pathways were enriched in the HGW-SARA group than in the LGW-CON group but not in the HGC-SARA group. Ten coenriched pathways related to HGW-SARA vs. LGW-CON and HGC-SARA vs LGW-CON were identified (Fig. S2A–C). Among them, only the gene expression patterns of the influenza A and IL-17 signalling pathways were almost opposite in these 2 comparisons (Fig. S2D and S2E). GSEA revealed that the expression of genes involved in the IL-17 signalling pathway was increased in the HGW-SARA group but decreased in the HGC-SARA group (Fig. S2D–S2F). The expression of the proinflammatory chemokine *CCL20* and the proinflammatory cytokine *PTGS2* was increased in the HGW-SARA group, but the expression of the proinflammatory genes *IL-17A*, *IL-17B*, *IL-17F*, and *FOS* was decreased in the HGC-SARA group (Fig. S3A and S3B).

**Fig. 2:**
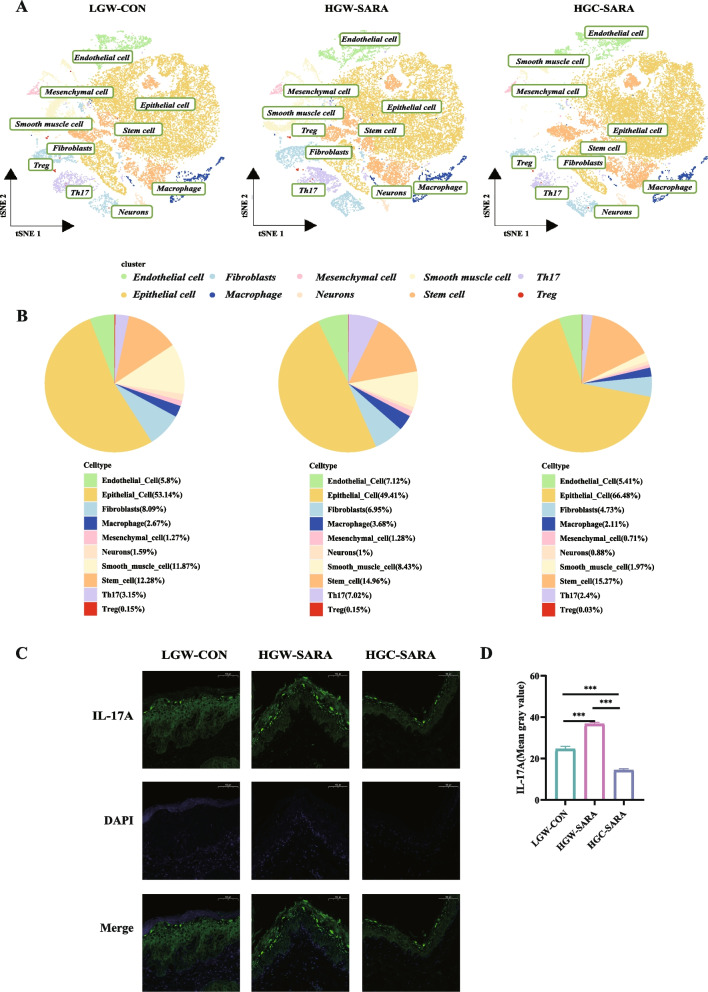
Various compositions of cell types in the rumen epithelium. **A** The t-SNE plot of 10 cell types in the rumen epithelium. **B** Ratios of different cell types in LGW-CON, HGW-SARA and HGC-SARA goats. **C** Immunofluorescence showing that the expression of IL-17A. **D** Statistics of IL-17 expression levels among the three groups

Endothelial cells, epithelial cells, fibroblasts, macrophages, mesenchymal cells, neurons, smooth muscle cells, stem cells, Th17 cells and regulatory T (Treg) cells were identified in the rumen epithelial samples (Fig. 2A). Among these cells, a comparative assessment of the cellular composition in the three groups revealed an increased proportion of Th17 cells, specifically in HGW-SARA goats (7.02%), a marked increase over LGW-CON (3.15%), and HGC-SARA (2.40%) (Fig. 2B). Given that IL-17A serves as an established biomarker for Th17 cells, immunofluorescence assays were carried out to map their distribution and quantify their expression levels (Fig. 2C, 2D).

### Altered rumen microbial community in dairy goats when SARA occurred

**Fig. 3:**
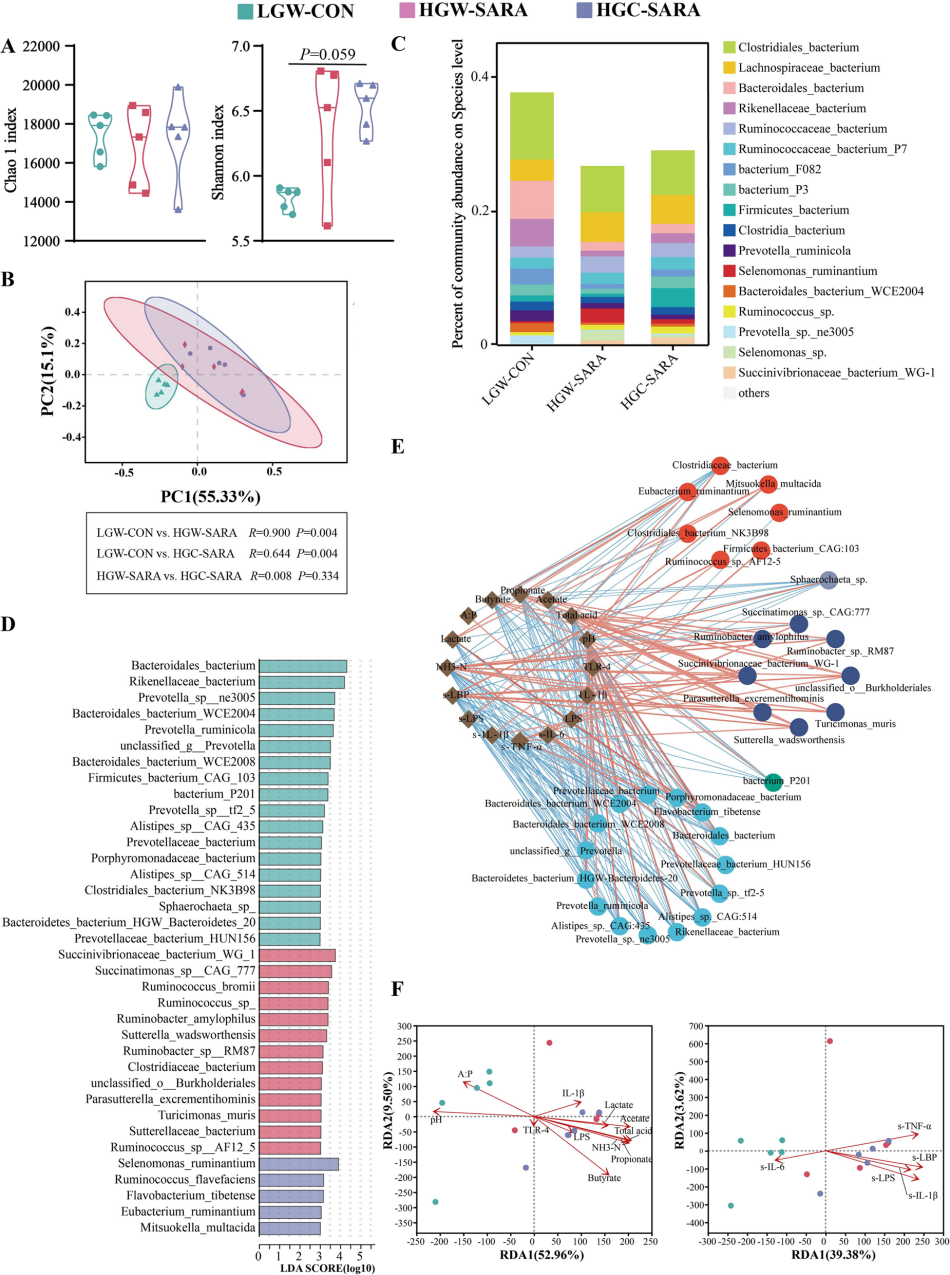
Identification of the characteristics of the ruminal microbiome and its function in different diets. **A**, **B** The α diversity and β diversity differences in the rumen fluid microbiome according to the Chao1 index and Shannon index (**A**) and PCoA of the rumen fluid microbiome (**B**) based on the NR database. **C** Rumen microbiome composition at the species level. **D** The significantly different species among LGW-CON, HGW-SARA and HGC-SARA goats identified by LEfSe analysis with LDA > 3 and *P* < 0.05. **E** Spearman correlation analysis between differential species and phenotypes related to fermentation and inflammation. The red line indicates a positive correlation, but the blue line indicates a negative correlation, with all the associations being significant. **F** RDA showing the relationships between species from different groups and fermentation and inflammatory phenotypes

The Shannon index of the HGC-SARA group was greater than that of the LGW-CON group (Fig. 3A; *P* = 0.059). β diversity analysis of the identified species revealed a significantly altered microbiome between the SARA goats (HGW-SARA and HGC-SARA) and the healthy goats (LGW-CON) (Fig. 3B). The SARA goats presented increased *Ruminococcus* and decreased *Prevotella* (Fig. 3C). Furthermore, LEfSe analysis revealed that the abundances of *Bacteroides bacterium*, *Bacteroidales bacterium WCE2004*, *Bacteroidetes bacterium HGW Bacteroidetes 20*, *Prevotella sp ne3005*, *Prevotella ruminicola*, *Prevotella sp tf2 5* and others, from the genera *Prevotella* and *Bacteroidales*, were decreased in SARA goats (Fig. 3D). And the abundance of species from the *Ruminococcus* were significantly increased in the SARA groups (Fig. 3D). Compared with those in the LGW-CON group, the microbial carbohydrate metabolism abilities were significantly weakened in the SARA goats, especially the relative abundances of cellulose and hemicellulose enzymes, such as GH26, GH51, GH10, GH92, and GH130 (FDR value < 0.05, LDA value > 3), whereas the lysozymes and LPS synthesis enzymes, such as GH24, GH25 and GT8, significantly increased (Fig. S4A–4B).

Correlation analysis indicated that the rumen microbiota might be involved in inducing rumen epithelial inflammation when SARA occurred in dairy goats. Of these, the *Ruminococcus sp. AF.12–5*, and *Mitsuokella multacida* which upregulated in SARA occurrence groups, were significantly positively correlated with the serum IL-1β, LBP, IL-6, and LPS contents (Fig. 3E). The RDA results indicated that the ruminal IL-1β and LPS concentrations; and the serum LPS, LBP, IL-1B, and TNF-α concentrations were significantly positively related to the ruminal microbiome composition of the 2 SARA groups (Fig. 3F).

### Microbial tryptophan metabolism participated in the regulation of ruminal inflammation

**Fig. 4:**
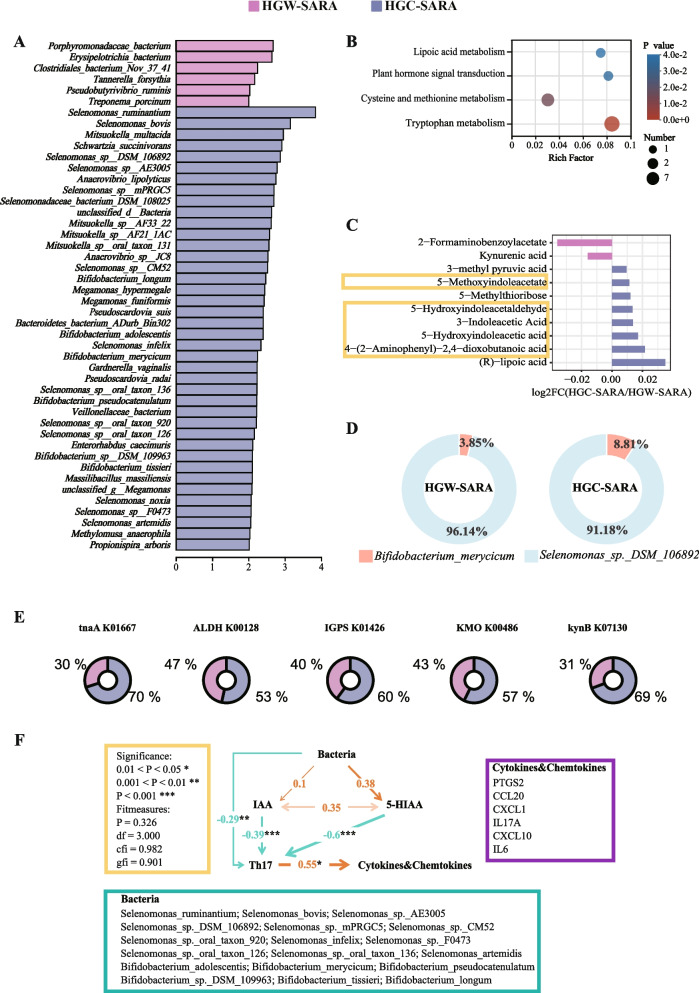
Bacteria enriched in HGC-SARA modulates the occurrence of inflammation via tryptophan derives IAA and 5-HIAA. **A** Differences in the abundances of ruminal bacteria in HGC-SARA and HGW-SARA goats identified by LEfSe analysis with LDA > 2, *P* < 0.05. **B** Pathway enrichment analysis was conducted on the significantly differentially abundant metabolites between HGW-SARA and HGC-SARA. **C** Ten metabolites with significant differences in pathways were shown in **B**. **D** The contribution analysis of species to tryptophan metabolism. **E** The KEGG function annotation along with the selection of genes encoding enzymes involved in tryptophan metabolism. **F** SEM revealed that the number of bacteria in the genera *Selnomonas* and *Bifidobacterium* increased in HGC-SARA goats, as did IAA, 5-HIAA, the number of Th17 cells as well as cytokines and chemokines. The numbers adjacent to the arrows are indicative of the effect size of the relationship: orange and red indicate positive regulation, and green indicates negative regulation

Integrated transcriptomics and snRNAseq revealed significantly downregulated expression of IL-17 signalling pathway genes such as *PTGS2*, *CCL20*, and *IL-17A* (*P* < 0.05) and reduced Th17 cell differentiation activity in the HGC-SARA group compared with the HGW-SARA group (*P* < 0.05) (Fig. S5A–S5B). Concurrently, in the HGC-SARA group, the relative abundances of those species from the *Selenomonas* and *Bifidobacterium* genera were significantly greater than those in the goats from the HGW-SARA group (LDA > 2, *P* < 0.05) (Fig. 4A). Metabolomics uncovered 146 differentially abundant metabolites between the HGW-SARA and HGC-SARA groups, with tryptophan metabolism being a key enriched pathway (*P* < 0.05) (Table S7 and Fig. 4B). Notably, five of ten tryptophan-derived metabolites, such as 3-indoleacetic acid (IAA) and 5-hydroxyindoleacetic acid (5-HIAA), were upregulated in HGC-SARA (*P* < 0.05) (Fig. 4C). The species and functional contribution analysis indicated that the two main species contributing to tryptophan metabolism were *Bifidobacterium merycicum* (3.85% for HGW-SARA and 8.81% for HGC-SARA) and *Selenomonas sp. DSM 106892* (96.14% for HGW-SARA and 91.18% for HGC-SARA) (Fig. 4D). Microbial enzyme analysis highlighted the increased expression of tryptophan-metabolizing genes such as *tnaA*, *ALDH*, *IGPS*, *KMO* and *kynB* in HGC-SARA (Fig. 4E).

SEM analysis revealed that bacteria from the genera *Selenomonas* and *Bifidobacterium* that were upregulated in the HGC-SARA group could modulate the tryptophan derivatives IAA and 5-HIAA to significantly negatively regulate the number of Th17 cells in the ruminal epithelium, subsequently affecting the secretion of inflammatory cytokines and chemokines (Fig. 4F). We compared tryptophan metabolism genes in the rumen epithelium and identified a total of 52 genes, but they did not show significant differences between the HGW-SARA and HGC-SARA groups (Fig. S6). Taken together, these data indicated that the differences in Th17 cell differentiation and IL-17 signaling pathways caused by differences in tryptophan metabolism only depend on microbial metabolism.

## The tryptophan metabolism profile in the ruminal microbiome

**Fig. 5:**
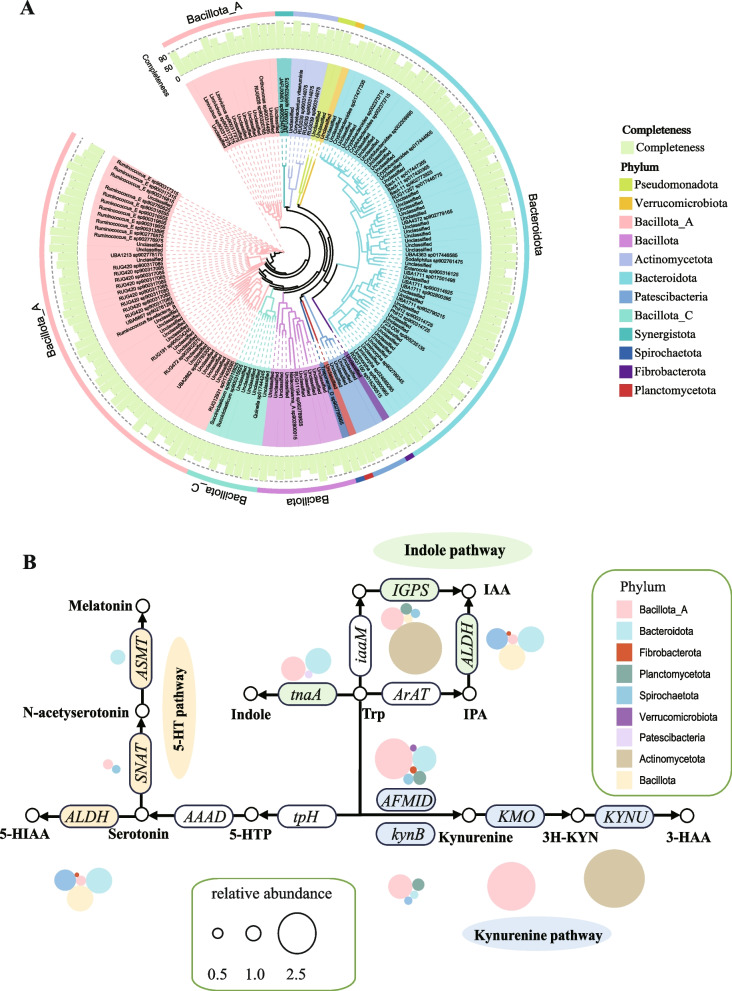
Profiles of MAGs based on metagenomic binning and the KEGG enzymes annotation about tryptophan metabolism. **A** Phylogenetic analysis of 154 MAGs of bacteria; from outside to inside, the phylum to which each MAG belongs, the degree of completeness, and the species-level annotation are shown. **B** The Tryptophan metabolism pathway. The circle packing represents the phylogenetic origin of the corresponding functional role at the phylum level, and circle size represents the relative abundance

The annotation of ruminal MAGs profiles was also performed. The bacterial clusters were dominated by *Bacteroidota* and *Bacillota A* in the phylogenetic tree (Table S8 and Fig. 5A). Three pathways involved in tryptophan metabolism were identified. In the indole pathway, tryptophan was directly transferred to indole via *tnaA* and synthesized as IAA, with high relative abundances of *ALDH* and *IGPS* (total relative abundance > 5) (Fig. 5B). In the 5-HT pathway, a high relative abundance of *ALDH* could helped to produce high concentrate of 5-HIAA. Meanwhile, a very low relative abundance of *SNAT* and *ASMT* (total relative abundance < 2) were identified in ruminal microbiome (Fig. 5B). In the kynurenine pathway, 3-HAA can be synthesized de novo by tryptophan via *AFMID*, *kynB*, *KMO* and *KYNU* with a relative abundance > 2. However, *KMO* and *KYNU* were only be identified in Bacillota A and Actinomycitota, respectively (Fig. 5B).

### The rumen microbial role in the occurrence of SARA and epithelial inflammation

**Fig. 6:**
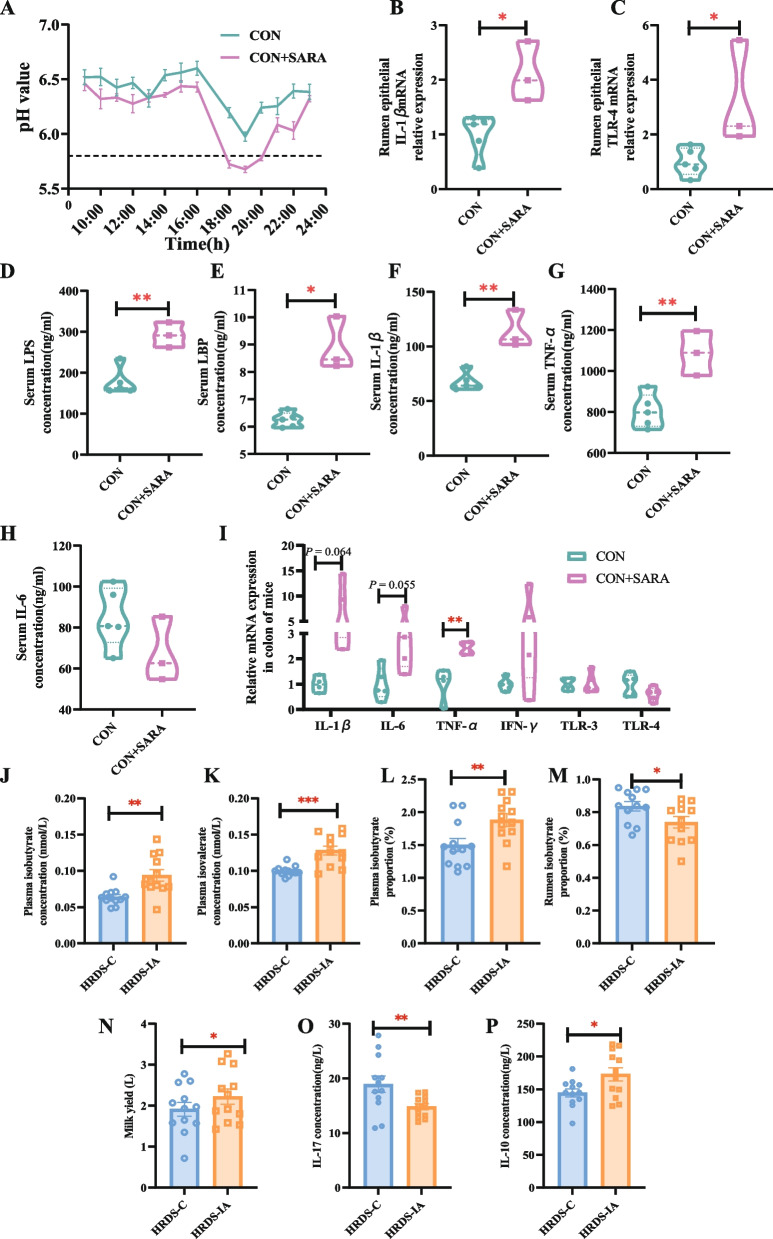
RMT verified the microbial role in SARA and inflammation occurrences and the phenotype of IAA gavaged goats. **A** Dynamic pH variation of dairy goats 14 h after morning feeding on the 14th day after RMT. **B**, **C** Relative expression of rumen epithelial IL-1β (**B**) and TLR-4 (**C**). **D**–**H** The concentrations of serum LPS (**D**), LBP (**E**), IL-1β (**F**), TNF-α (**G**) and IL-6 (**H**) in dairy goats. **I** Relative expression of inflammatory cytokines in the colons of the mice. **J**–**M** Differential isobutyrate (**J**) and isovalerate (**K**) concentration or isobutyrate (**L**) and isovalerate (**M**) proportions of ruminal fluids and plasma between HRDS-C and HRDS-IA. (N) The comparison of milk yield between HRDS-C and HRDS-IA. **O**–**P** The comparison of IL-17 (**O**) and IL-10 (**P**) in plasma between the HRDS-C and HRDS-IA groups

To identify the microbial role, the ruminal contents of HGW-SARA goats were transplanted into another 3 healthy goats. Compared with those in the CON group, which did not receive RMT, the pH level in the rumen of goats in the CON + SARA group remained below 5.8 for more than 3 h (Fig. 6A). Furthermore, after RMT, the relative expression of the ruminal epithelial genes IL-1β and TLR-4 in receiving goats was significantly greater than that in the LGW-CON group (Fig. 6B, 6C). The serum LPS, LBP, IL-1β, and TNF-α concentrations were also significantly greater in the CON + SARA group than in the LGW-CON group (Fig. 6D–H). Moreover, the RMT mouse model was also further used to detect the role of the rumen microbiome in the occurrence of gastrointestinal epithelial inflammation, which was also associated with increased mRNA expression of proinflammatory factors (Fig. 6I).

### Supplementation with 3-indoleacetic acid was beneficial for preventing SARA and increasing milk yield

Compared with those in HRDS-C dairy goats, the concentrations of isobutyrate and isovalerate as well as the proportion of isobutyrate in the plasma significantly increased, whereas the proportions of isobutyrate significantly decreased in the rumens of HRDS-IA goats (Fig. 6J–M). Furthermore, the milk yield was significantly greater in HRDS-IA goats than in HRDS-C goats on the 42nd day (Fig. 6N). The measurement of cytokines in the plasma revealed that the level of IL-17 significantly decreased and that of IL-10 significantly increased in HRDS-IA goats (Fig. 6O, 6P).

## Discussion

To meet the nutritional demand of dairy goats to ensure high milk production, high-concentrate diets were provided to dairy goats in China to increase the ruminal rumen digestion starch content [21, 50]. Our research confirmed that a high-concentration diet, especially an increased level of ruminal starch, leads to increased risks of SARA in dairy goats [51, 52]. SARA has been widely shown to affect ruminant feed intake, growth performance, whole gastrointestinal digestibility, and rumen inflammation [2, 53]. On the basis of these findings, the present study is one of the few studies that further focused on goats that suppress ruminal inflammation when ruminal SARA occurs. Moreover, our study systemically identified alterations in the ruminal microbiome, metabolome, host ruminal transcriptome, and ruminal single-nucleus transcriptome in response to high-RDS feeding and its associated mechanisms in regulating the occurrence of SARA and ruminal inflammation and its suppression.

Our study first revealed that the relative abundances of cellulose and hemicellulose enzymes, such as GH26 and GH51, were significantly decreased in SARA goats, whereas the lysozymes and LPS synthesis enzymes, including GH24 and GH25, were significantly increased in SARA goats. The increased lysozymes and LPS synthesis enzymes induce the lysis of bacteria involved in the cellulolytic process and accumulation of LPS [54, 55], which have been suggested to be the key inducers of ruminal inflammation [56, 57].Furthermore, it has been reported that the variation in the expression of the Toll-like receptor genes *TLR2* and *TLR4* in the rumen epithelial wall significantly changed in steers with differential susceptibility to subacute ruminal acidosis [58, 59]. Our rumen epithelial transcriptome analysis also revealed that the TLR/MyD88-NFκB pathway-mediated ruminal inflammation process occurs in dairy goats with SARA. Through the detection of the expression levels of IL-1B and TLR4 in the rumen epithelium and the concentrations of LPS, LBP, IL-1B and TLR4 in the blood, it was proven that SARA could indeed cause rumen epithelial inflammation and systemic inflammation. In addition, we discovered that Th17 cell differentiation and the IL-17 signalling pathway, which have been demonstrated to regulate intestinal inflammation in mammals [60, 61], can be key regulatory modules of the ruminal inflammation process. Mechanistically, inflammatory Th17 cells concomitantly activate cytokines such as IL-17, IL-22, TNF-α, and IFN-γ, which further activate the TLR/MyD88-NFκB pathway, thereby instigating an inflammatory response in the gastrointestinal epithelium [62, 63]. Hence, our findings lead to the speculation that inflammation in SARA goats can be initiated through the promotion of Th17 cell differentiation, the activation of the IL-17 signalling pathway, and the subsequent release of inflammatory cytokines to induce ruminal inflammation.

In the present study, we observed that ruminal Th17 cell differentiation, IL-17 signalling, and inflammation were inhibited in HGC-SARA goats and that the relative abundances of those species from the *Selenomonas*, *Mitsuokella*, and *Bifidobacterium* genera significantly increased. In previous studies, *Selenomonas* was suggested to participate in cellulose degradation and lactate consumption [64], and *Mitsuokella* was suggested to be involved in phytase production and carbohydrate metabolism [65, 66]. Moreover, as the most important previously suggested probiotics, several species of *Bifidobacterium* have been widely identified, and their tryptophan metabolites IAA and 5-HIAA may be involved in the regulation of Th17 cell differentiation homeostasis [67]. On the basis of these identified differential species between the HGW-SARA and HGC-SARA groups, we found that microbial tryptophan metabolism was increased in goats without ruminal inflammation under SARA conditions. We focused on *Bifidobacterium merycicum* and *Selenomonas sp. DSM 106892*, two bacteria that played an important role in ruminal microbial tryptophan metabolism. Research indicates that tryptophan metabolism encompasses pathways such as kynurenine, serotonin, and indole metabolism [68]. In the indole metabolic pathway, gut microbes can directly convert tryptophan into indole and its derivatives, including IAA and indole propionic acid [69]. These specific species from *Selenomonas*, *Mitsuokella*, and *Bifidobacterium* can inhibit the transcriptional activity of RORγt by producing tryptophan-indole metabolites, inhibiting the differentiation of gastrointestinal Th17 cells and the IL-17-mediated gastrointestinal inflammation process and maintaining the homeostasis of the gut microbiota and internal environment [70, 71]. The process of host tryptophan metabolism has also been widely shown to contribute to maintaining immune homeostasis and intestinal barrier function [68]. However, in this study, transcriptome and snRNA analyses of ruminal epithelium tissues did not reveal significant changes in host genes involved in the regulation of tryptophan metabolism.

Among those related differentially abundant metabolites, IAA can inhibit ruminal inflammation by suppressing Th17 cell differentiation and IL-17 signalling activation. According to previous studies, reduced IAA and indole derives could be responsible for an increased Th17 cell response and polarization, as well as CD4^+^IL-17A^+^ cell enrichment [12]. Furthermore, IAA was confirmed to play an important role in rumen epithelial development [68]. Mechanistically, indoles and their derivatives, including IAA, can act as ligands for the aryl hydrocarbon receptor (AhR) [72]. Once activated, AhR translocates into the nucleus, where it forms a heterodimer with the AhR nuclear translocator (ARNT) [73]. This complex influences the transcription of downstream genes, such as G protein-coupled receptor 15 (GPR15), promoting epithelial cell proliferation [74], maintaining intestinal homeostasis, and enhancing nutrient absorption. In addition, the activation of AhR can promote intestinal and rumen epithelial cell proliferation by regulating the AhR-AKT/CREB axis or the AhR-Wnt/β-catenin signalling pathway [68, 74]. Additionally, upon activation, AhR can competitively bind to RORγt, inhibiting the differentiation of inflammatory Th17 cells [75]. However, it is widely believed that Th17 cells in the gastrointestinal epithelium migrate from the peripheral circulation, making it challenging to isolate them from the rumen epithelium or rumen organoids solely to verify the inhibitory effect of IAA on Th17 cells differentiation and the secretion of the pro-inflammatory factor IL-17 [76]. On the other hand, separating cultured Th17 cells out of the limitation of rumen epithelium, lead to unreliable result. On the basis of these findings, we further validated our results via another dairy goats feeding trial and tested the role of IAA in preventing SARA and rumen epithelial inflammation caused by Th17 cells under high-RDS diets. These results show that dietary supplementation of goats with IAA can help reduce ruminal inflammation and promote VFAs absorption, which can prevent SARA occurrence and further increase dairy goats’ milk yield. Overall, the most important finding in the present study is that ruminal microbiome-derived IAA inhibits ruminal inflammation in subacute ruminal acidosis goats by suppressing Th17 cell and IL-17 signalling pathway activation and promoting VFAs absorption and increasing milk yield.

## Conclusion

This study unveiled that after SARA was induced by high-concentrate feeding, the rumen homeostasis was disrupted and rumen fiber degradation capacity of dairy goats reduced but LPS synthesis capacity increased, and inflammation of the rumen epithelium was observed. However, the ruminal microbiome from *Bifidobacterium* and *Selenomonas* and bacterial 3-indoleacetic acid are pivotal in mitigating ruminal epithelial inflammation during SARA in dairy goats. This could potentially be attributed to the modulation of ruminal Th17 cell proportions and the inhibition of IL-17 signalling pathways.

## Supplementary Information

Supplementary Material 1: Figure S1 The experiments design of SARA dairy goats model construction, ruminal microbiota transplantation to dairy goats and mice, as well as the IAA gavage.

Supplementary Material 2: Figure S2 Transcriptome analysis of rumen epithelial tissues to investigate inflammation in SARA goats. (A-B) The significantly differential KEGG enrichment pathways of DEGs between LGW-CON and HGW-SARA (A) and between LGW-CON and HGC-SARA (B). (C-E) Ten common differential pathways of LGW-CON vs HGW-SARA and LGW-CON vs HGC-SARA. (F) GSEA revealed differences in the IL-17 signalling pathway.

Supplementary Material 3: Figure S3 Volcano maps exhibiting the differential expression genes. (A) 110 genes significantly increased and 71 genes significantly decreased in HGW-SARA goats comparing to LGW-CON goats. (B) 125 genes significantly increased and 231 genes significantly decreased in HGC-SARA goats comparing to LGW-CON goats. The marked genes all played role in IL-17 signalling pathway.

Supplementary Material 4: Figure S4 The differences of microbial function and CAZymes. (A) Differential microbial functions identified by LEfSe analysis on the basis of the KEGG database with LDA >3 and *P* < 0.05. (B) Differences in microbial enzymes based on CAZy database.

Supplementary Material 5: Figure S5 Transcriptome and snRNA of rumen epithelial tissues indicating that inflammation occurs in HGW-SARA goats but not in HGC-SARA goats. (A) Immune-related KEGG pathways enriched via DEGs between HGW-SARA and HGC-SARA samples and six genes in the IL-17 signalling pathway were significantly upregulated in HGW-SARA goats (P < 0.05, fold change ≥ 2 or ≤ 0.5). (B) Compared with the HGC-SARA group, the top 30 enriched KEGG metabolic pathways that the HGW-SARA group presented significant upregulation in Th17 cells.

Supplementary Material 6: Figure S6 The volcano map showed the DEGs between HGW-SARA and HGC-SARA in tryptophan metabolism.

Supplementary Material 7: Table S1 Ingredients and nutrient composition of the low and high-concentrate diet on a dry matter (DM) basis. Table S2 Ingredients and nutrient composition of the 50% concentrate diet on a dry matter (DM) basis. Table S3 Ingredients and nutrient composition of the 60% concentrate diet on a dry matter (DM) basis. Table S4 Ingredients of the diet and antibiotic feeding the mice. Table S5 Primers used in quantitative PCR analysis. Table S6 The body weight comparisons of dairy goats in 3 groups. Table S7 The differential metabolites between HGC-SARA and HGW-SARA dairy goats. Table S8 The 163 MAGs with medium to high quality.

## Data Availability

All the data generated or analysed for this study are included in this paper and the supplementary material. The metagenome data, RNA-seq data, metabolomics data and single-nucleus RNA-seq data were deposited into the China National Genebank (https://db.cngb.org/cnsa/) under accession number CNP0006226.
