# Correction: Altered ruminal microbiome tryptophan metabolism and their derived 3-indoleacetic acid inhibit ruminal inflammation in subacute ruminal acidosis goats

**DOI:** 10.1186/s40168-025-02305-5

**Published:** 2025-12-06

**Authors:** Xiaodong Chen, Jingyi Xu, Lei Zhang, Bingxuan Xie, Jianrong Ren, Jinghui He, Tao Liu, Qingqing Liu, Yachen Dong, Xiaolong He, Junhu Yao, Shengru Wu

**Affiliations:** 1 College of Animal Science and Technology, Northwest A&F University, Yangling, Shaanxi 712100 China; 2 Key Laboratory of Livestock Biology, Northwest A&F University, Yangling, Shaanxi 712100 China; 3 College of Animal Science and Technology, Ningxia University, Yinchuan, 750021 China; 4 Shanghai Majorbio Biopharm Technology Co. Ltd, Shanghai, 201318 China

**Correction: Microbiome 13, 215 (2025)**

**https://doi.org/10.1186/s40168-025-02202-x**

Following publication of the original article [1], the author reported that in Figure 1F, the HE staining image of LGW-CON was misused, and now is updating to the correct paraffin section result. Below is the incorrect Figure 1. 
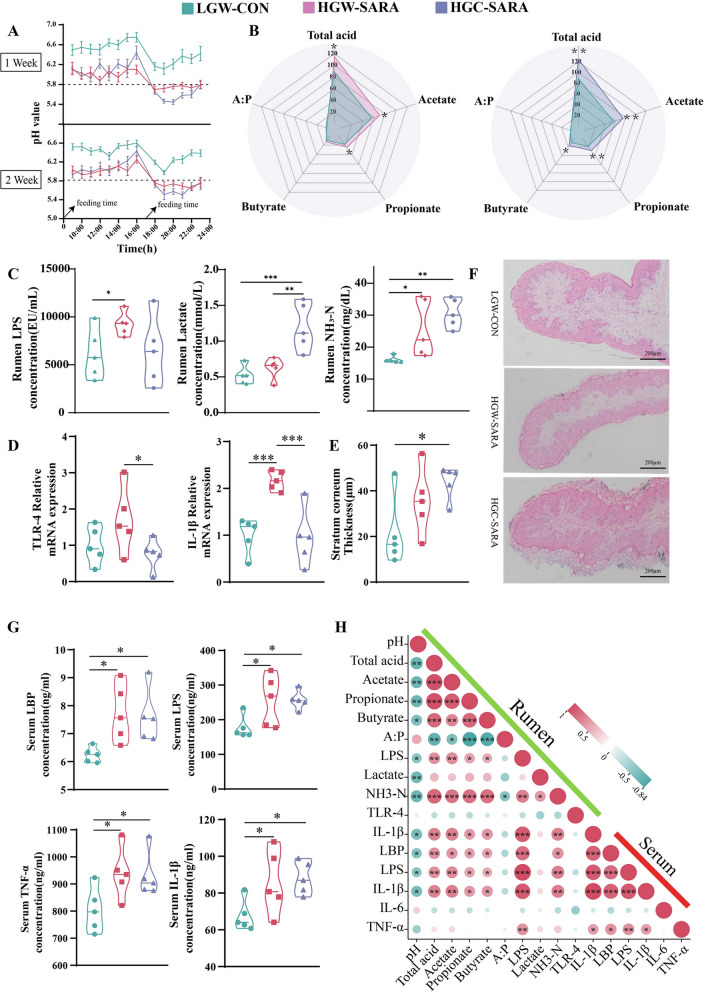


Below is the correct Figure 1. 
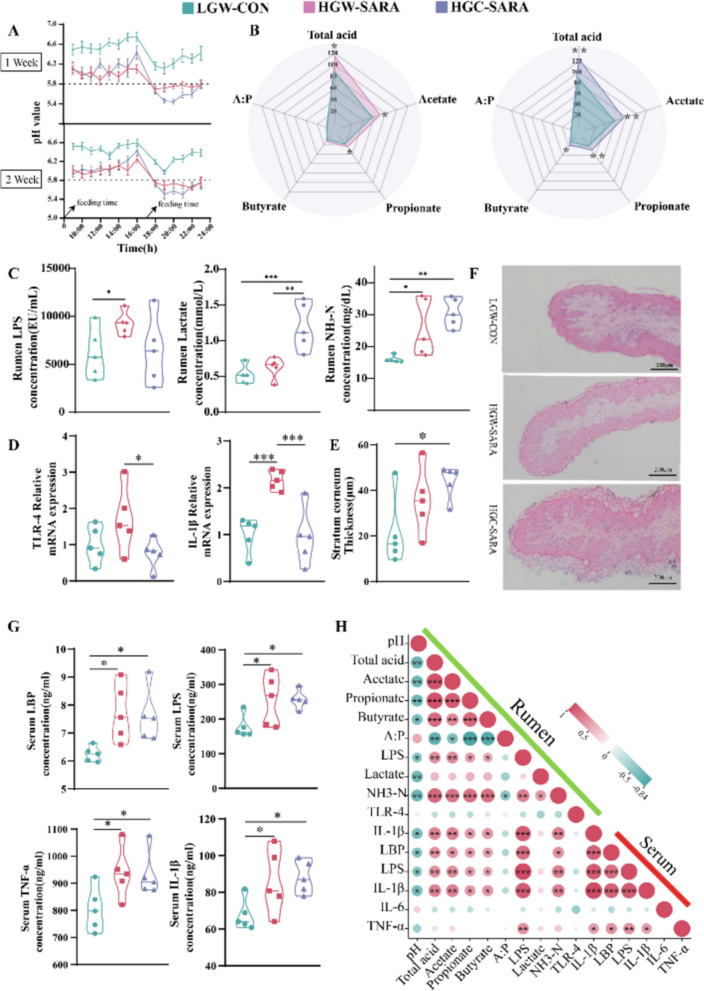


Secondly, the figure citation of Fig. 6P was missing. The text should be “The measurement of cytokines in the plasma revealed that the level of IL-17 significantly decreased and that of IL-10 significantly increased in HRDS-IA goats (Fig. 6O, 6P).”

The original article has been updated.
