# Supplementary material for: Altered ruminal microbiome tryptophan metabolism and their derived 3-indoleacetic acid inhibit ruminal inflammation in subacute ruminal acidosis goats
Source: Microbiome. 2025 Oct 23;13:215. doi: 10.1186/s40168-025-02202-x (PMC12548289; doi:10.1186/s40168-025-02202-x)
Supplement: Supplementary file 2 — Supplementary Material 1: Figure S1 The experiments design of SARA dairy goats model construction, ruminal microbiota transplantation to dairy goats and mice, as well as the IAA gavage. [file 40168_2025_2202_MOESM1_ESM.pdf]

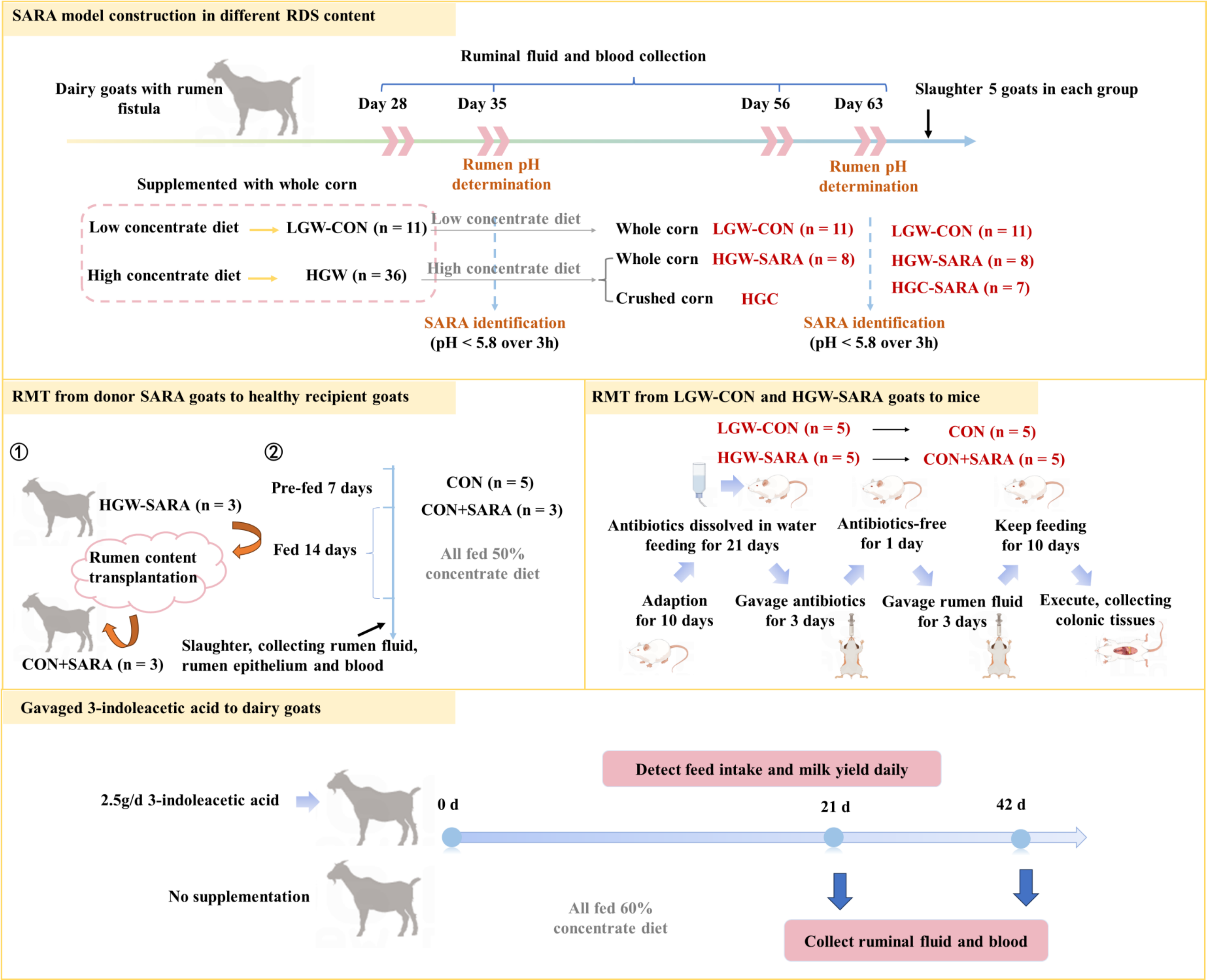
SARA model construction in different RDS content
Ruminal fluid and blood collection
Dairy goats with rumen fistula
Day 28
Day 35
Day 56
Day 63
Slaughter 5 goats in each group
Rumen pH determination
Rumen pH determination
Supplemented with whole corn
Low concentrate diet
LGW-CON (n = 11)
High concentrate diet
HGW (n = 36)
Low concentrate diet
High concentrate diet
Whole corn
LGW-CON (n = 11)
Whole corn
HGW-SARA (n = 8)
Crushed corn
HGC
LGW-CON (n = 11)
HGW-SARA (n = 8)
HGC-SARA (n = 7)
SARA identification (pH < 5.8 over 3h)
SARA identification (pH < 5.8 over 3h)
RMT from donor SARA goats to healthy recipient goats
①
②
HGW-SARA (n = 3)
Rumen content transplantation
CON+SARA (n = 3)
Pre-fed 7 days
Fed 14 days
CON (n = 5)
CON+SARA (n = 3)
All fed 50% concentrate diet
Slaughter, collecting rumen fluid, rumen epithelium and blood
RMT from LGW-CON and HGW-SARA goats to mice
LGW-CON (n = 5)
CON (n = 5)
HGW-SARA (n = 5)
CON+SARA (n = 5)
Antibiotics dissolved in water feeding for 21 days
Antibiotics-free for 1 day
Keep feeding for 10 days
Adaption for 10 days
Gavage antibiotics for 3 days
Gavage rumen fluid for 3 days
Execute, collecting colonic tissues
Gavaged 3-indoleacetic acid to dairy goats
Detect feed intake and milk yield daily
2.5g/d 3-indoleacetic acid
0 d
21 d
42 d
No supplementation
All fed 60% concentrate diet
Collect ruminal fluid and blood
