# Supplementary material for: Altered ruminal microbiome tryptophan metabolism and their derived 3-indoleacetic acid inhibit ruminal inflammation in subacute ruminal acidosis goats
Source: Microbiome. 2025 Oct 23;13:215. doi: 10.1186/s40168-025-02202-x (PMC12548289; doi:10.1186/s40168-025-02202-x)
Supplement: Supplementary file 3 — Supplementary Material 2: Figure S2 Transcriptome analysis of rumen epithelial tissues to investigate inflammation in SARA goats. (A-B) The significantly differential KEGG enrichment pathways of DEGs between LGW-CON and HGW-SARA (A) and between LGW-CON and HGC-SARA (B). (C-E) Ten common differential pathways of LGW-CON vs HGW-SARA and LGW-CON vs HGC-SARA. (F) GSEA revealed differences in the IL-17 signalling pathway. [file 40168_2025_2202_MOESM2_ESM.pdf]

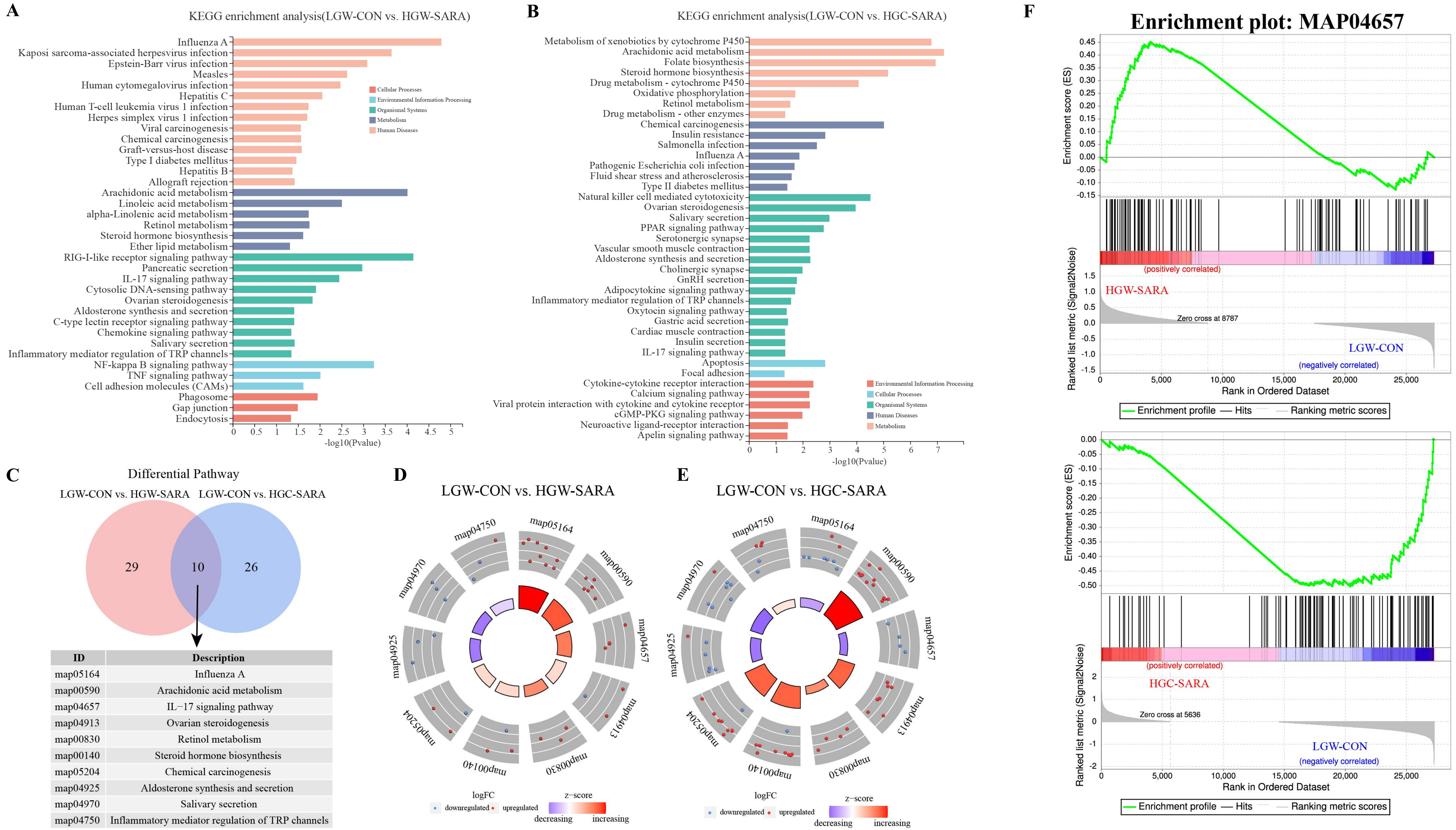

ID	Description
map05164	Influenza A
map00590	Arachidonic acid metabolism
map04657	IL−17 signaling pathway
map04913	Ovarian steroidogenesis
map00830	Retinol metabolism
map00140	Steroid hormone biosynthesis
map05204	Chemical carcinogenesis
map04925	Aldosterone synthesis and secretion
map04970	Salivary secretion
map04750	Inflammatory mediator regulation of TRP channels
