# Supplementary figures and images for: Altered ruminal microbiome tryptophan metabolism and their derived 3-indoleacetic acid inhibit ruminal inflammation in subacute ruminal acidosis goats
Source: Microbiome. 2025 Oct 23;13:215. doi: 10.1186/s40168-025-02202-x (PMC12548289; doi:10.1186/s40168-025-02202-x)

**A**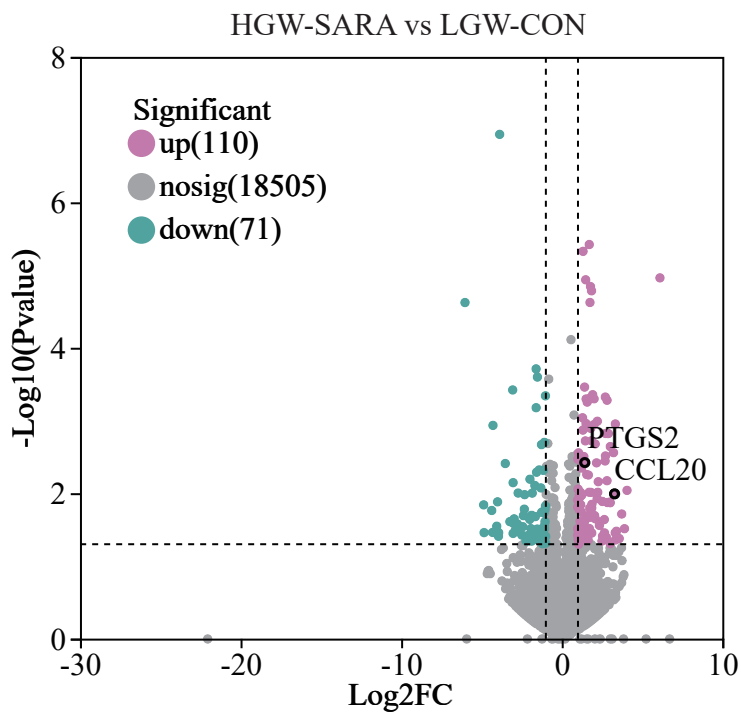**B**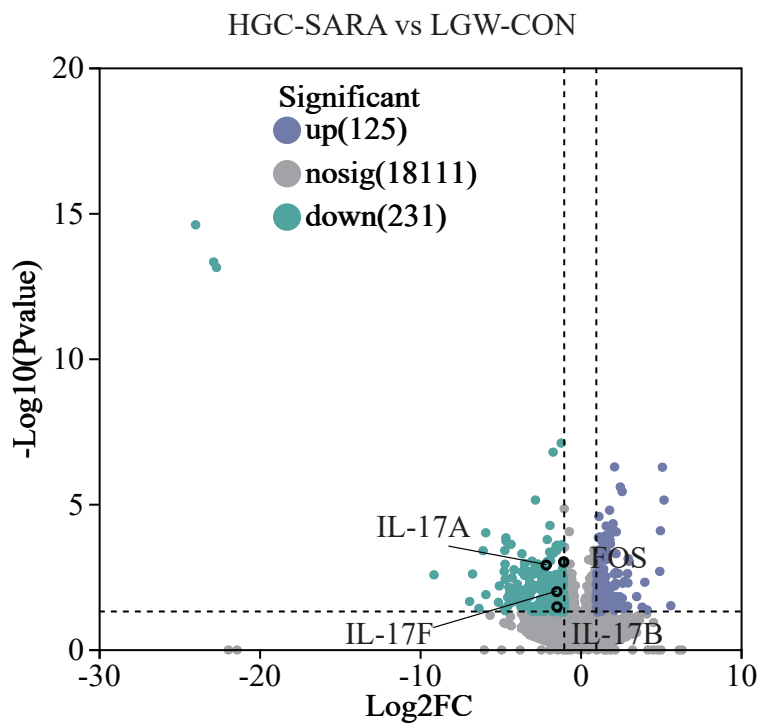

Supplement: Supplementary file 4 — Supplementary Material 3: Figure S3 Volcano maps exhibiting the differential expression genes. (A) 110 genes significantly increased and 71 genes significantly decreased in HGW-SARA goats comparing to LGW-CON goats. (B) 125 genes significantly increased and 231 genes significantly decreased in HGC-SARA goats comparing to LGW-CON goats. The marked genes all played role in IL-17 signalling pathway. [file 40168_2025_2202_MOESM3_ESM.pdf]

A

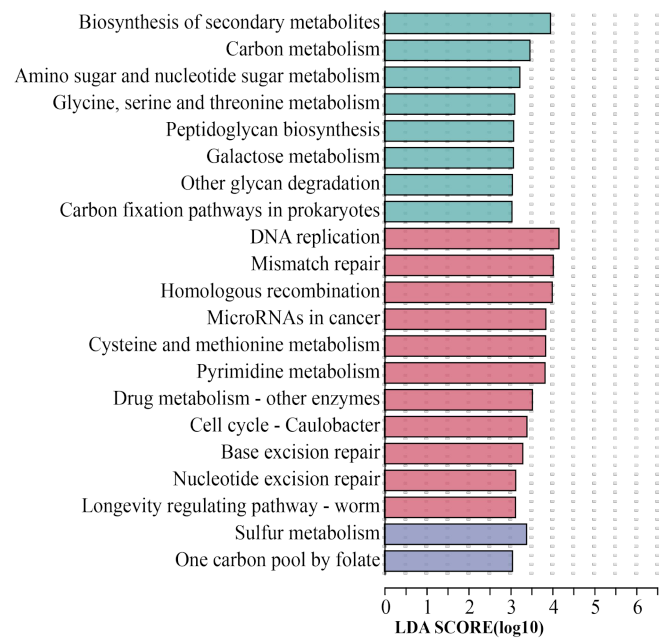

B

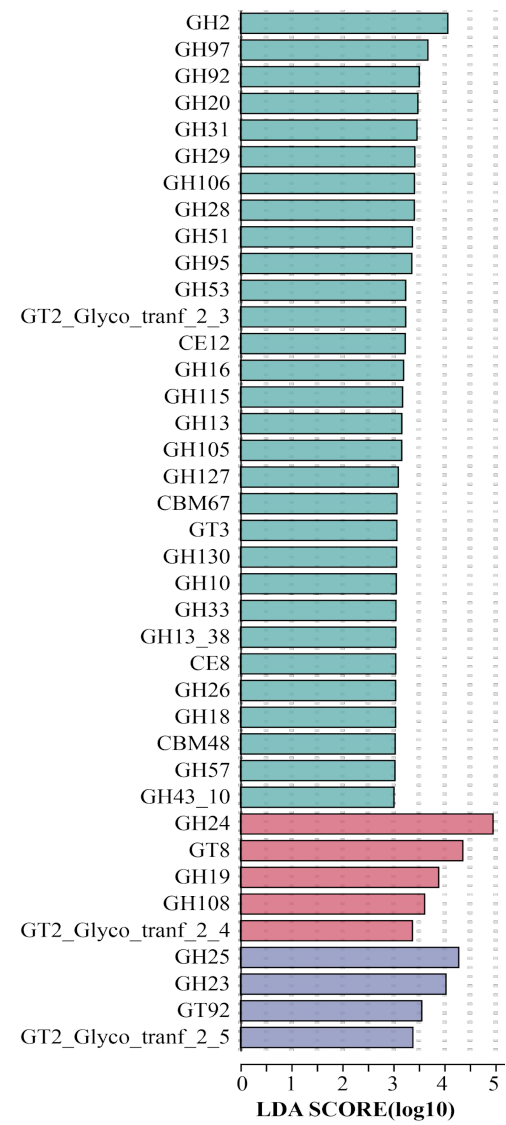

Supplement: Supplementary file 5 — Supplementary Material 4: Figure S4 The differences of microbial function and CAZymes. (A) Differential microbial functions identified by LEfSe analysis on the basis of the KEGG database with LDA >3 and P < 0.05. (B) Differences in microbial enzymes based on CAZy database. [file 40168_2025_2202_MOESM4_ESM.pdf]

A

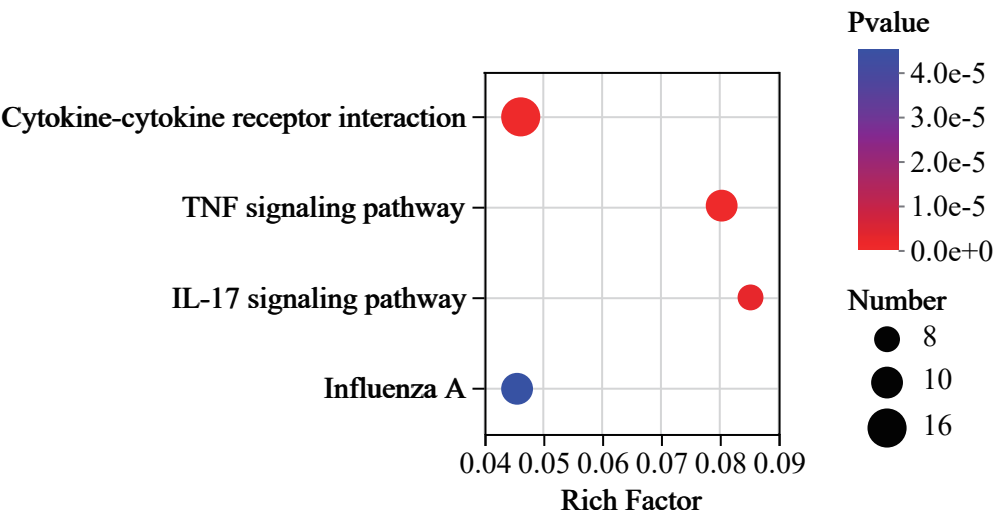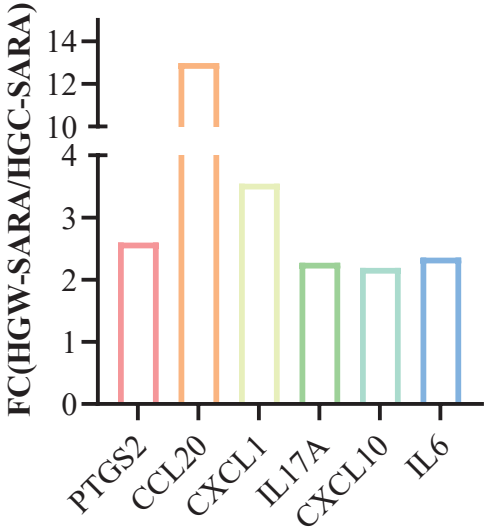

B

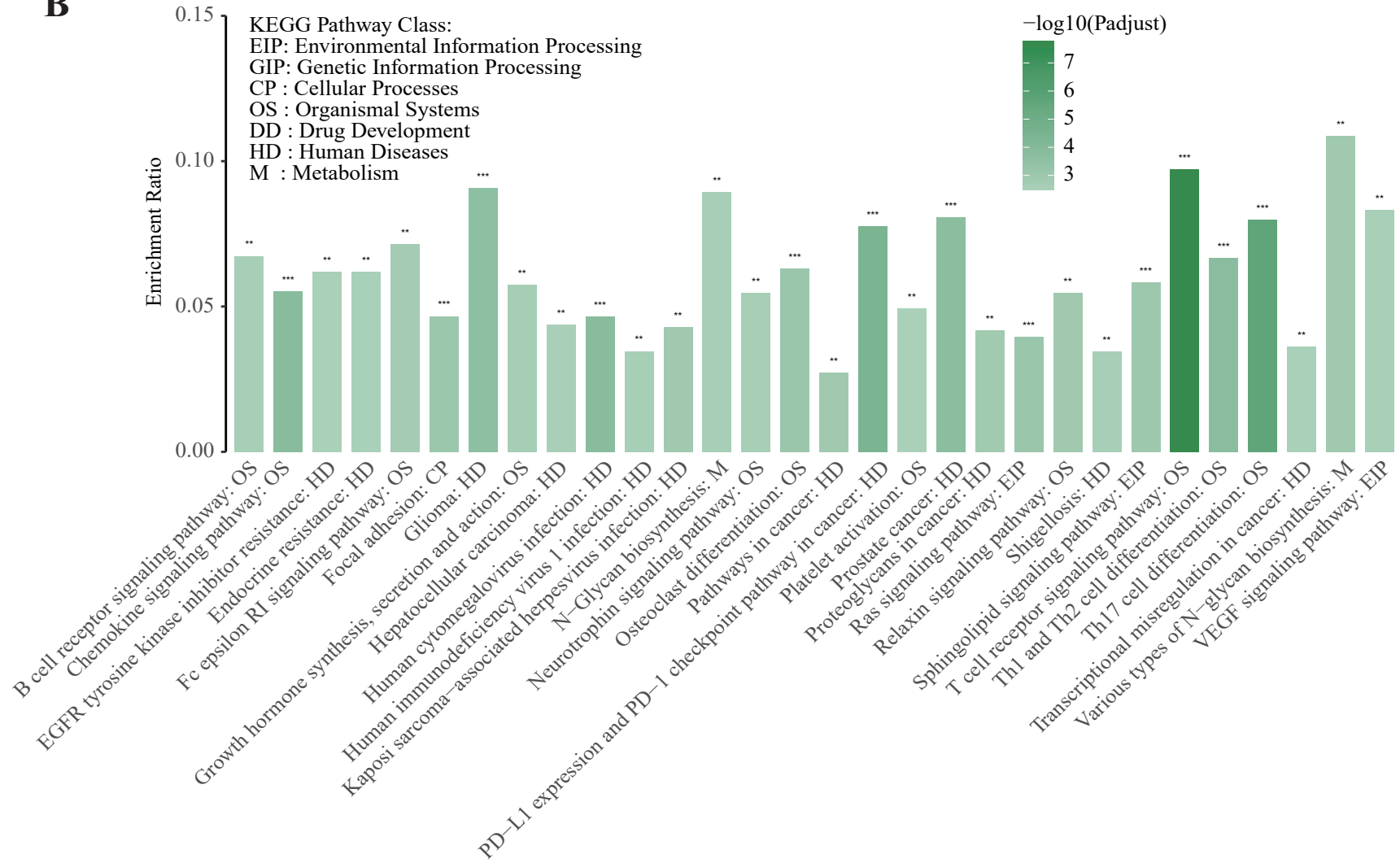

Supplement: Supplementary file 6 — Supplementary Material 5: Figure S5 Transcriptome and snRNA of rumen epithelial tissues indicating that inflammation occurs in HGW-SARA goats but not in HGC-SARA goats. (A) Immune-related KEGG pathways enriched via DEGs between HGW-SARA and HGC-SARA samples and six genes in the IL-17 signalling pathway were significantly upregulated in HGW-SARA goats (P < 0.05, fold change ≥ 2 or ≤ 0.5). (B) Compared with the HGC-SARA group, the top 30 enriched KEGG metabolic pathways that the HGW-SARA group presented significant upregulation in Th17 cells. [file 40168_2025_2202_MOESM5_ESM.pdf]

Total DEGs: 52

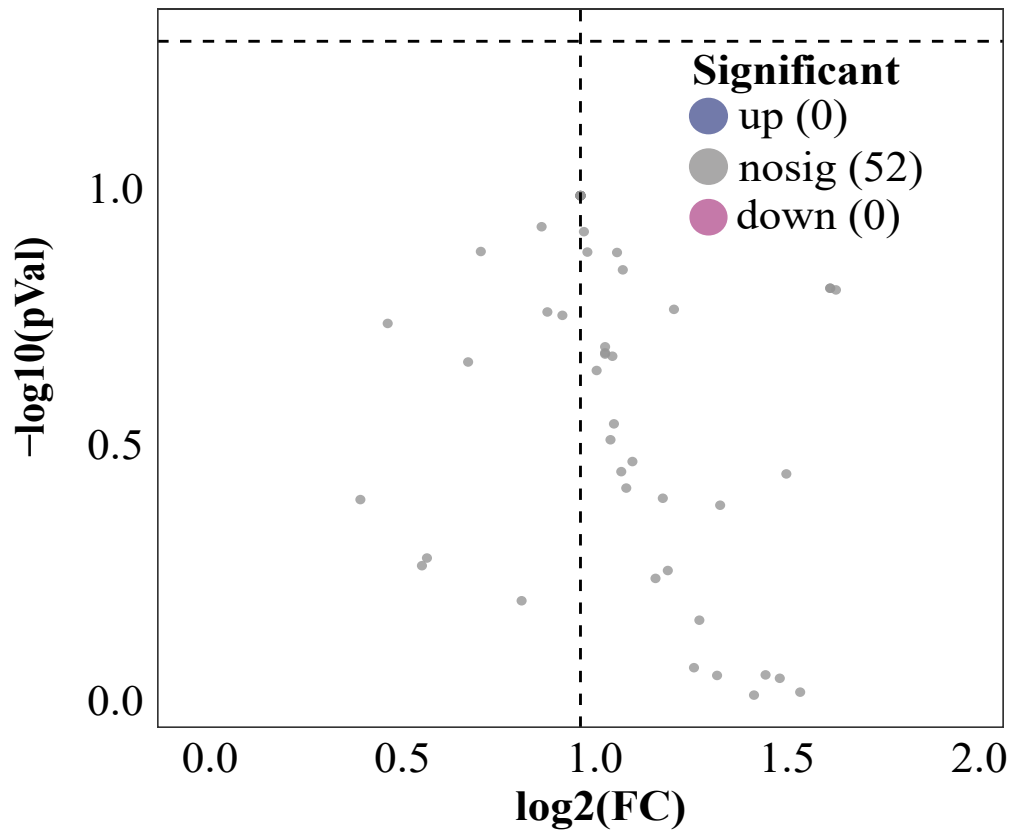

Supplement: Supplementary file 7 — Supplementary Material 6: Figure S6 The volcano map showed the DEGs between HGW-SARA and HGC-SARA in tryptophan metabolism. [file 40168_2025_2202_MOESM6_ESM.pdf]
